# An Atypical Presentation of Creutzfeldt-Jakob Disease as a Stroke Mimic: Experience From an Irish Tertiary Center

**DOI:** 10.7759/cureus.43066

**Published:** 2023-08-07

**Authors:** Hassan Al Taie, Nikhil Vasandani, Armon Nasehi, Tom O'Malley

**Affiliations:** 1 Internal Medicine, Mayo University Hospital, Castlebar, IRL; 2 Surgery, Royal College of Surgeons in Ireland, Dublin, IRL

**Keywords:** creutzfeldt-jakob disease, ireland, stroke, subacute spongiform encephalopathy, prion protein, prion diseases

## Abstract

Sporadic Creutzfeldt-Jakob Disease (sCJD) is a rare neurodegenerative prion disease that presents with symptoms of rapid neuropsychiatric decline including dementia, behavioural abnormalities, and loss of higher cortical function. Patients commonly present with rapidly progressive neuromotor symptoms such as ataxia and myoclonus. Very few cases of CJD have been reported in which the patient initially presents with stroke symptoms such as hemiparesis as their primary presenting symptom.

We present a case of a 56-year-old male who initially presented to the stroke unit with waxing and waning left-sided weakness and a non-corresponding ipsilateral left-sided acute parietal infarct on diffusion-weighted MRI. Over four weeks, his condition progressively worsened with declining cognitive function, motor dysfunction, sphincter dysfunction, and eventual death.

## Introduction

Sporadic Creutzfeldt-Jakob disease (sCJD) is the most commonly noted prion disease within humans, accounting for approximately 85% of all cases of CJD [1,2]. Despite this, CJD is a relatively rare disease with a worldwide incidence of approximately one case per million annually [3].

The pathophysiology of the condition involves the accumulation of misfolded neural proteins known as prions (PrPSc) mainly within the thalamus and cerebral cortex of the brain [4]. Most cases present with rapid onset behavioural changes, memory impairment, emotional lability, and loss of higher cortical function demonstrated by symptoms such as disinhibition and apraxia [5]. Other common presenting symptoms include neuromotor symptoms such as ataxia or myoclonus [6,7].

CJD is a uniformly fatal condition. All individuals affected experience a rapid decline in their neurological symptoms, with eventual akinetic mutism. Eighty percent of those affected die within a year of diagnosis. Very few cases of CJD have initially presented with stroke-like symptoms such as hemiparesis, leading to the initial consideration of stroke as a primary diagnosis [8-10].

Magnetic resonance imaging (MRI) is the most useful imaging modality for detecting CJD. Findings most commonly include hyperintense lesions in the corpus callosum, the caudate nucleus, the superior parietal lobe, and the frontal gyrus [11,12]. Imaging findings are often paired with characteristic periodic sharp wave complexes (PSWC) seen on an electroencephalogram (EEG) and detection of 14-3-3 protein in the cerebrospinal fluid (CSF) [13,14]. Since 2017, the detection of CSF RT-QuIC for sporadic sCJD prion protein has been included in the diagnostic criteria for CJD, with a sensitivity of 92% and specificity of 100% for detecting CJD [15,16]. Here, we present a case of CJD in a 56-year-old male who presented with left-sided periodic weakness and identification of an ipsilateral acute infarct on MRI, indistinguishable from an acute cerebral infarction. It is important to consider CJD as a differential when dealing with patients presenting with atypical stroke histories alongside incongruous imaging findings seen in radiology. 

## Case presentation

A 56-year-old male with no family history of neurological disease presented to the emergency department with a six-week history of progressively worsening left upper limb paresthesia, paralysis, and left lower limb paresthesia. The patient had no remarkable past medical history and was a non-smoker and non-drinker. His occupational history was also unremarkable. Given that this patient presented newly to the emergency department FAST positive with stroke-like symptoms, this clinically was regarded as a new, acute-onset stroke. No record of the patient's baseline neurological status was documented, and he had not presented to any other hospitals or primary care physicians at the start of symptom onset. 

The patient was vitally stable upon arrival at the emergency department. On examination, he was normotensive with a blood pressure of 124/84 mmHg, his heart rate was 75 beats per minute, his respiratory rate was 16 breaths per minute, and he had a temperature of 37.2 degrees Celsius. The patient had a normal BMI.

Neurological examination revealed intact cranial nerves including normal visual fields. The patient had marked impairment in coordination of the left side with past-pointing in the upper and lower limbs. He had left-sided upper limb weakness, left-sided upper limb, and lower limb paresthesia indiscernible to sharp touch. Gait pattern and general clinical examination were otherwise normal.

**Figure 1:**
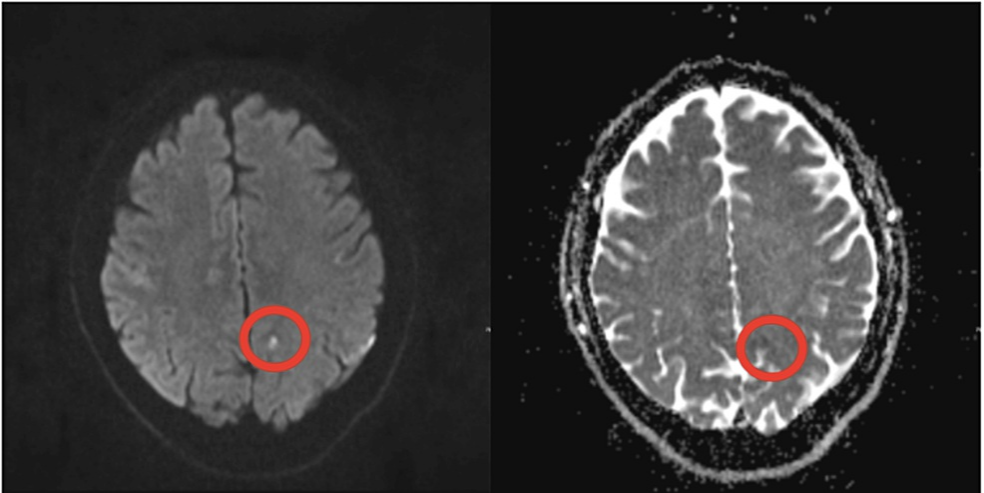
Axial B1000 DWI MRI (left) and corresponding ADC map (right) reveal a tiny less than 5 mm diameter of acute diffusion restriction in the left posterior cerebral hemisphere most in keeping with a tiny acute focus of infarction (labelled) DWI: Diffusion-weighted imaging; ADC: apparent diffusion coefficient

Routine laboratory investigations conducted were all normal. As part of the FAST-positive workup for queried stroke, the patient underwent initial contrast and non-contrast computed tomography (CT) scans of the brain, carotid arteries, and intracranial CT angiogram which were normal and revealed no evidence of carotid stenosis or dissection. The patient underwent subsequent vascular ultrasonography of the carotids which showed no significant stenosis. Normal findings on CT ultimately warranted a subsequent MRI of the brain. An axial B1000 diffusion-weighted image (DWI) and corresponding apparent diffusion coefficient (ADC) mapping revealed a tiny less than 5 mm diameter of acute diffusion restriction in the left posterior cerebral hemisphere most in keeping with a tiny acute focus of infarction (Figure 1). This finding, although implicative of acute infarction, was ipsilateral to the side of the clinical symptoms. Due to MRI findings of infarction being incongruous with the clinical symptoms, a definitive diagnosis of stroke could not be reached. A transesophageal echocardiogram was performed which also was normal. The patient was commenced on a once-daily dose of 300 mg aspirin for 14 days given the single finding of a silent acute cortical infarct on MRI imaging.

Over the following ten days, the patient became progressively worse with declining cognitive function, behavioural issues, and myoclonic jerking of the left upper limb. The patient began to exhibit signs of disinhibition and over the next 72 hours developed a severe disregard for bladder and bowel control. The patient failed to maintain basic personal hygiene due to being incontinent of urine and faeces. He required increased overall nursing care needs, became bed-bound, and required full-time care.

Blood samples were taken for Lyme disease, human immunodeficiency virus (HIV), syphilis, and hepatitis serology. All investigations were negative. The patient underwent a follow-up MRI which revealed persistence of the same <5 mm small acute infarct in the left parietal lobe with no changes from prior imaging. A lumbar puncture was performed on day 21 and toxoplasmosis infection was excluded.

A sample of CSF was collected and sent to the lab. Samples collected were also sent to the Irish National Creutzfeldt-Jakob disease Surveillance Unit as CJD was now being suspected as a potential differential diagnosis.

Initial cell and protein count indicated increased protein levels. CSF blood, glucose, and gram stain were all negative. The patient deteriorated further and now required full-time care. He became aphonic and obtunded. He was transferred under the care of the neurology service in the hospital. An EEG was performed which demonstrated periodic sharp wave complexes occurring maximally in the left centrotemporal region. The EEG finding was suggestive of a diagnosis of prion disease. The patient died one week later, and on the day of his death, the National CJD Surveillance Unit confirmed the cerebrospinal fluid analysis was positive for RT-QuIC indicating sporadic CJD.

## Discussion

Our case highlights a presentation of CJD. The absence of characteristic neuroimaging findings paired with initial stroke-mimicking symptoms and MRI findings of an acute infarct raised suspicions of a cerebrovascular event.

Neuroimaging plays a vital role in the diagnosis of CJD. Classical findings include hyperintense lesions in the corpus callosum, the caudate nucleus, and the superior parietal lobe on DWI, T2, and fluid-attenuated inversion recovery (FLAIR) sequencing [17,18]. Many observed cases show increased lesions confined to the pulvinar thalamic nucleus; this is known as the pulvinar sign [18]. Stroke-mimicking presentations of CJD are very rare and reported cases showed typical subcortical hyperintensities on imaging [8,9]. Serial MRI in these cases is noted to have shown progressive neurological changes with initial abnormal hyperintensities in the caudate putamen and subcortical areas, eventually progressing to generalized atrophy and ventricular dilatation at end-stage disease [13]. This specific case is quite unusual as serial MRIs showed the persistence of a small 3 mm focus of hyperattenuation visualised posteriorly in the left fronto-occipital region. Despite the clinical deterioration of the patient, MRI findings were unchanged, and the single left-sided site of acute infarct remained persistent. The probability of ischemic stroke in this case was low considering MRI findings being ipsilateral to the clinical signs exhibited by the patient. Left-sided stroke symptoms with subsequent left-sided ischemic changes on neuroimaging are extremely rare with only a handful of cases observed in the literature [19].

Periodic sharp wave complexes are the most classical EEG finding for CJD and are seen in two third of the patients diagnosed with sporadic CJD [14]. Such changes are often seen in the middle to later stages of the disease, whereas early disease shows non-specific diffuse changes on EEG [14]. The presence of the 14-3-3 protein in the CSF has been found to be a sensitive and specific marker for sporadic CJD. This alongside serial MRIs and EEG is sufficient to exclude other differential diagnoses [15,16]. Despite this, brain biopsy remains the gold standard for definitive diagnosis of CJD [13].

The stroke-mimicking nature of the initial presentation, in this case, raised clinical suspicion for a potential cerebral ischemic event. However, the absolute lack of any stroke risk factors, unilateral MRI findings, and rapid clinical deterioration raised the possibility of an infectious or rare neurological disorder instead. Moreover, the raised protein in the initial CSF sample with the exclusion of other toxic or metabolic causes quickly made CJD a likely diagnosis. A high index of suspicion is required to have reached the diagnosis in this case. A stroke mimic is atypical in CJD, and this hindered the standard path towards reaching the diagnosis. Unfortunately, the patient progressively deteriorated in this case. Despite the uniformly fatal nature of CJD, early lumbar puncture and CSF sampling would have rapidly reduced the time taken to reach a diagnosis. Through earlier diagnosis, the ceiling of care would be determined and thus have made a positive impact on the limited quality of life. Earlier detection allows for rapid involvement of the palliative care team and the commencement of comfort measures, as well as improved social planning for both the patient and their family at the end of life. We hope that this case will raise awareness of the atypical presentations of CJD, specifically its tendency to rarely present as a stroke mimic.

## Conclusions

In cases of patients presenting with clinical signs and symptoms of a stroke with subsequent ipsilateral neurological findings on MRI, CJD should be included as a differential diagnosis. Additionally, a lumbar puncture, not routinely recommended in stroke patients, should be performed early to aid in the early detection of CJD in order to confirm the diagnosis and optimise the ceiling of care.
